# Revealing the Yeast Diversity of the Flor Biofilm Microbiota in Sherry Wines Through Internal Transcribed Spacer-Metabarcoding and Matrix-Assisted Laser Desorption/Ionization Time of Flight Mass Spectrometry

**DOI:** 10.3389/fmicb.2021.825756

**Published:** 2022-02-09

**Authors:** Juan Carbonero-Pacheco, Jaime Moreno-García, Juan Moreno, Teresa García-Martínez, Juan Carlos Mauricio

**Affiliations:** Department of Agricultural Chemistry, Edaphology and Microbiology, Agrifood Campus of International Excellence CeiA3, University of Córdoba, Córdoba, Spain

**Keywords:** flor biofilm, biological aging, Sherry wine, ITS-metabarcoding, MALDI-TOF MS, *Saccharomyces cerevisiae*, non-*Saccharomyces*

## Abstract

Flor yeast velum is a biofilm formed by certain yeast strains that distinguishes biologically aged wines such as Sherry wine from southern Spain from others. Although *Saccharomyces cerevisiae* is the most common species, 5.8 S-internal transcribed spacer (ITS) restriction fragment length polymorphism analyses have revealed the existence of non-*Saccharomyces* species. In order to uncover the flor microbiota diversity at a species level, we used ITS (internal transcribed spacer 1)-metabarcoding and matrix-assisted laser desorption/Ionization time of flight mass spectrometry techniques. Further, to enhance identification effectiveness, we performed an additional incubation stage in 1:1 wine:yeast extract peptone dextrose (YPD) before identification. Six species were identified: *S. cerevisiae*, *Pichia manshurica*, *Pichia membranifaciens*, *Wickerhamomyces anomalus*, *Candida guillermondii*, and *Trichosporon asahii*, two of which were discovered for the first time (*C. guillermondii* and *Trichosporon ashaii*) in Sherry wines. We analyzed wines where non-*Saccharomyces* yeasts were present or absent to see any potential link between the microbiota and the chemical profile. Only 2 significant volatile chemicals (out of 13 quantified), ethanol and ethyl lactate, and 2 enological parameters (out of 6 quantified), such as pH and titratable acidity, were found to differ in long-aged wines. Although results show a low impact where the non-*Saccharomyces* yeasts are present, these yeasts isolated from harsh environments (high ethanol and low nutrient availability) could have a potential industrial interest in fields such as food microbiology and biofuel production.

## Introduction

Sherry wines are distinctive wines elaborated in the southern Spanish areas of Jerez–Xerez–Sherry, Montilla–Moriles, and Condado de Huelva. In these regions, Sherry wines are made using a traditional method termed “criaderas-solera,” which involves a dynamic biological aging process carried out by flor yeast, which are specific strains of *Saccharomyces cerevisiae*. These strains can form a biofilm in the wine–air interphase referred to as flor or velum. The biological aging begins after spontaneous stabilization of the wine once alcoholic and malolactic fermentations are concluded (Peinado and Mauricio, 2009; Moreno-García et al., 2013).

The criaderas–solera system consists of numerous rows (criaderas) of 600 L oak barrels with wine in various stages of aging. The rows are numbered from the floor to the top where the first lies on the ground, so called “solera,” and contains the most aged wine; the second row above is the “first criadera” and subsequently the second, third, etc. Before the criadera–solera system, the wine is kept in ∼2,000 L clay jars known as “sobretablas.” Glycerol and volatile acidity levels decrease, while acetaldehyde increases as the wine approaches solera (Moreno-García et al., 2013).

Flor formation is induced by the lack of non-fermentable carbon sources that trigger the migration of yeast cells to the wine surface where oxygen is more abundant and the biological aging takes place. During this process, yeast cells oxidize ethanol to acetaldehyde and acetate, while glycerol is gradually catabolized (Martínez et al., 1998). From a molecular perspective, flor formation is regulated by the expression of genes such as *FLO11*, which results in higher cell-surface hydrophobicity and multicellular aggregate formation (Legras et al., 2016; Moreno-García et al., 2018).

Until now, several identification techniques have been aimed to characterize the flor microbiota. Flor yeast strains *S. cerevisiae beticus*, *cheresiensis*, *montuliensis*, and *rouxii* have been identified in Sherry wines based on their ability to metabolize different carbon and nitrogen sources (Martínez et al., 1997; Mesa et al., 1999). However, the limitations of this technique such as long execution time or low identification accuracy drove scientists to use molecular methodologies. By the amplification of the ITS1 (internal transcribed spacer 1)–ITS4 regions in the Jerez–Xerez–Sherry solera wine flor, Ruiz-Muñoz et al. (2020) revealed the nine genotypes of *S. cerevisiae* using microsatellite typing. The same authors also reported the occurrence of non-*Saccharomyces* species after molecular identification. Similar results were obtained by amplifying the 5.8S-ITS and interdelta regions to identify *S. cerevisiae* strains in Jura (France) from the isolates of flor samples, revealing that flor biofilms could be formed by single or multiple strains (David-Vaizant and Alexandre, 2018). Another limitation in the characterization of the flor velum microbiota is the scarce capability of some microorganisms to proliferate under laboratory conditions, thus hindering their detection.

The study of wine microbiota by next-generation sequencing (NGS) has ushered in a new era of biodiversity surveillance without the need of microorganism cultures between sampling and identification, enabling a high-throughput analysis of complex microbial communities *via* short amplicons (Bokulich et al., 2012; Berbegal et al., 2019). For these reasons, we hypothesize that the use of NGS techniques, such as internal transcribed spacer (ITS)-metabarcoding, using the ITS1 will reveal a higher diversity of yeasts in solera barrels. The ITS1 region of the eukaryotic ribosomal cluster has features that allow for wide taxonomic coverage and has been recognized as a suitable barcode region for the species-level identification of fungal organisms (Usyk et al., 2017).

Besides NGS, microbial identification through matrix-assisted laser desorption/ionization time of flight mass spectrometry (MALDI-TOF MS) has recently gained popularity due to its cheaper costs and its application in many different areas such as medicine, food, military science, and ecological research. Microbial identification using MALDI-TOF MS compares peptide mass fingerprint (PMF) from the unknown organism from an axenic culture, with the PMF entries in the database, or by comparing the mass of the biomarker in the unknown organism with the reference proteome database (Han et al., 2021). This technology has been widely used to identify yeasts from wine samples in recent years (Usbeck et al., 2014; Kačániová et al., 2020; Zhang et al., 2021), being a cheap and feasible option for a rapid and accurate identification of *S. cerevisiae* and non-*Saccharomyces* isolates at the genus and species level (Gutiérrez et al., 2017).

In this study, we analyze for the first time the flor velum microbiota in criaderas–solera Sherry wine barrels in Montilla-Moriles by using novel culture-independent (ITS-metabarcoding) and -dependent techniques (MALDI-TOF MS). We also characterized the chemical profiles of the wine samples to see whether there were any possible correlations between the wine and the microbiota identified.

## Materials and Methods

### Flor Velum Sampling

**FIGURE 1:**
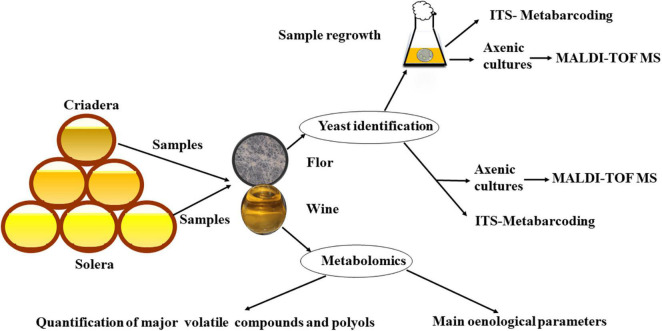
Workflow of material and methods/experimental design.

Flor velum samples were taken from 13 wine barrels at the Gracia winery in Montilla-Moriles region between September 2020 and April 2021 at various stages of the criaderas–solera system: 7 from solera and 6 from the first criadera. To collect flor yeast samples, a special sterile steel net was used. The net was sterilized by flame with 96% (v/v) ethanol before and after every sampling. The flor velum sampled was resuspended in 25 ml wine from same barrel in sterile tubes. Once in the laboratory, samples were treated in different ways (Figure 1). About 2 L of wine for each barrel were also sampled to quantify the oenological parameters according to the International Organisation of Vine and Wine (OIV) (2020) and to grow sampled yeasts in the laboratory.

To obtain higher biomass prior to identification, half of each sample was inoculated into a 300 ml media consisting of 1:1 yeast extract peptone dextrose (YPD) broth (1% yeast extract, 2% bacteriological peptone, 2% glucose) and wine in 500 ml Erlenmeyer flasks. This treatment was called “regrowth,” and the samples were incubated at 28°C and 175 rpm for 5 days and later under static conditions at 22°C for 5 days. The reason for this treatment was that untreated samples had a poor viability rate, due to the demanding conditions in the winery. Further, flor velum samples were cultivated at 22°C in wine from the same barrel in Erlenmeyer flasks to assess the development of the biofilm under laboratory conditions.

For ITS-metabarcoding identification, samples were directly processed, but for MALDI-TOF, in order to obtain isolates, the flor velum from 13 samples (7 from solera and 6 from first criadera) with and without regrowth were plated in YPD agar plates after serial dilutions and incubated for 5 days at 28°C. Then, 10 colonies randomly selected from each plate were isolated to obtain axenic cultures and subsequently identified by MALDI-TOF. Every sample was plated in triplicate.

### Yeast Identification by Internal Transcribed Spacer-Metabarcoding

Genomic DNA was extracted from 1 to 1.5 ml of flor velum resuspended in wine or YPD broth, containing yeasts by using a quick yeast genomic DNA extraction kit (Bio Knowledge Lab, S.L., Córdoba, Spain), following the guide provided by the manufacturer. The DNA from three samples (solera 1–2 and criadera 1) with and without regrowth were extracted.

The fungal ITS region was amplified using specific primers (ITS5-1737F and ITS2-2043R) with barcodes. All PCR reactions were carried out with Phusion^®^ High-Fidelity PCR Master Mix (New England Biolabs, Ipswich, MA, United States).

To visualize PCR amplification, an equal volume of 1 × loading buffer (with SYB green) and PCR products were loaded on 2% (w/v) agarose gel for detection. Samples with bright main strip between 400 and 450 bp were chosen for further experiments. PCR products were mixed at equal density ratios and then purified with Qiagen Gel Extraction Kit (Qiagen, Germany).

#### High-Throughput Sequencing

Purified amplicons were prepared for Illumina sequencing by constructing a library using NEBNext Ultra™ DNA Library Prep Kit for Illumina and quantified *via* Qubit and qPCR and were analyzed by Illumina platform. Amplicon was sequenced on Illumina paired-end platform to generate 250 bp paired-end raw reads. Additional sequencing was performed for samples with less than 100,000 raw tags.

#### Bioinformatic Analysis

Analysis was carried out using QIIME2 v2020.8 (Bolyen et al., 2019); the reads were denoised using DADA2 (Callahan et al., 2016), and the following processes were conducted: (1) trimming and truncating low-quality regions, (2) dereplicating the reads, and (3) chimera filtering.

After denoising, forward and reverse reads are merged into one sequence, dereplicated and assigned to an ID, considering them ASVs (amplicon sequence variants).

Amplicon sequence variants were organized in operational taxonomic units (OTUs) by using *de novo* clustering method from vsearch (Rognes et al., 2016). Clustering was performed at 97% identity to create 97% OTUs. OTUs were classified by taxon using an ITS1 region of the UNITE database (Nilsson et al., 2019) with vsearch (Rognes et al., 2016).

### Identification of Isolates by Matrix-Assisted Laser Desorption/Ionization Time of Flight Mass Spectrometry

A qualitative analysis of yeast isolates was performed with MALDI-TOF mass spectrometry (Bruker Daltonics, Bremen, Germany). A colony from each isolate was placed in 300 μl distilled water and 900 μl ethanol and vortexed until homogenization. Then, samples were pelleted at 13,000 rpm for 2 min, and the pellet was dried at room temperature. Lastly, 50 μl of 70% formic acid and 50 μl of acetonitrile were added to the pellet. Samples were centrifuged for 2 min at 13,000 rpm, obtaining a supernatant with proteins.

About 1 μl of supernatant was dried at room temperature in a matrix-assisted laser desorption/ionization (MALDI) plate, and each sample was coated with 1 L of HCCA matrix (α-cyano-4-hydroxycinnamic acid) prepared in a mixture of 50% acetonitrile and 2.5% trifluoroacetic acid. Samples were again dried at room temperature.

Dried samples were analyzed with MALDI-TOF/TOF “ULTRAFLEXTREME” (Bruker Daltonics, Bremen, Germany) equipment. Generated spectra were treated with MALDI Biotyper compass (MBT Compass; Bruker, Billerica, MA, United States) software, which calibrates the spectra and automatizes the measures and identifications before searching and matching the results. Obtained spectra were compared with reference profiles from the MBT Compass Library (Bruker). Scores ≥2.0 were used as a selection criterion for identifications at species level (Kačániová et al., 2020).

### Oenological Parameters

Wines with different microbiota were chemically analyzed in order to assess differences between them. The most important chemical analysis (CA) parameters from an oenological point of view are relative density, ethanol, titratable acidity, pH, volatile acidity, and free SO_2_. These parameters were quantified in accordance with the European Union Official Methods (International Organisation of Vine and Wine (OIV), 2020).

### Gas-Chromatographic Quantification of Major Volatile Compounds and Polyols

Considering the most abundant alcohols (methanol, 1-propanol, isobutanol, isoamylic, and 2-phenylethanol), 3 carbonyl compounds (acetaldehyde, 1,1-diethoxyethane, and acetoin), 3 ethyl esters (ethyl acetate, ethyl lactate, and ethyl succinate), 2 polyols (glycerol and 2,3-butanediol), and 13 wine aroma compounds were quantified by gas-chromatographic analysis (GCA) using the method of Peinado et al. (2004) and a model gas chromatograph (GC) 6890 from Agilent (Palo Alto, CA, United States). A CP-WAX 57 CB capillary column (60 m long × 0.25 mm i.d., 0.4 μm film thickness) from Varian (Palo Alto, CA, United States) was used, and 0.5 μl of wine or standard samples previously supplied with 1 ml of 1 g/L 4-methyl-2-pentanol as an internal standard solution were injected into the GC injector. A split ratio mode of 30:1 was set for sample dilution, and a flame ionization detector was used for chemical identification and quantification. An oven temperature program involving an initial temperature of 50°C (15 min), a 4°C/min ramp, and a final temperature of 190°C (35 min) was employed. The injector and detector temperatures were 270 and 300°C, respectively. The flowrate of carrier gas (helium) was initially set at 0.7 ml/min (16 min) and followed by a 0.2 ml/min ramp to the final value (1.1 ml/min), which was held for 52 min. Each compound was quantified using the response factor provided by standard solutions analyzed with the same methods as the wine samples. The chemical compounds used, the preparation of standards, and method validation was detailed by Peinado et al. (2004). Also, each compound was confirmed by using the Willey 7 N spectral library and a mass spectrometric detector model HP-5972-A from Agilent Technologies, coupled to the same GC equipment used for the quantification of standards and wine samples.

### Statistical Analysis

The multiple comparison analysis (MCA) for each chemical variable using the Bonferroni’s test at a confidence level of 95% (i.e., α = 0.05 significance level) was carried out in order to identify those variables showing significant differences in the wines sampled. MCA groups samples that show significant differences in homogeneous groups (HGs). Averages with different HGs show statistically significant differences at the 95.0% confidence level. The method currently being used to discriminate among the averages is Fisher’s least significant difference (LSD) procedure. With this method, there is a 5.0% risk of calling each pair of averages significantly different when the actual difference equals 0. The variables obtained in major volatile compounds and polyols analysis were subjected to a analysis of clusters (AC) in order to identify differences in the groups of wine based on the presence or absence of non-*Saccharomyces* yeasts in wine samples, AC with all variables obtained were analyzed too. This analysis identifies those samples showing a significant difference by means of the squared Euclidean distance as a measure of the proximity between two samples and the method of Ward as a clustering rule. The results from MCA were subjected to a principal component analysis (PCA), to obtain a small number of linear combinations of the most important variables in samples and group them in a biplot. Groups statistically analyzed were determined by their aging time (solera or criadera) and the presence or absence of non-*Saccharomyces* yeast in the velum of the barrel. All these described statistical analyses were performed with the Statgraphics^®^ Centurion XVIII Software Package from Stat Points Technologies, Inc. (Warrenton, VA, United States).

## Results

### Sample Treatment

**FIGURE 2:**
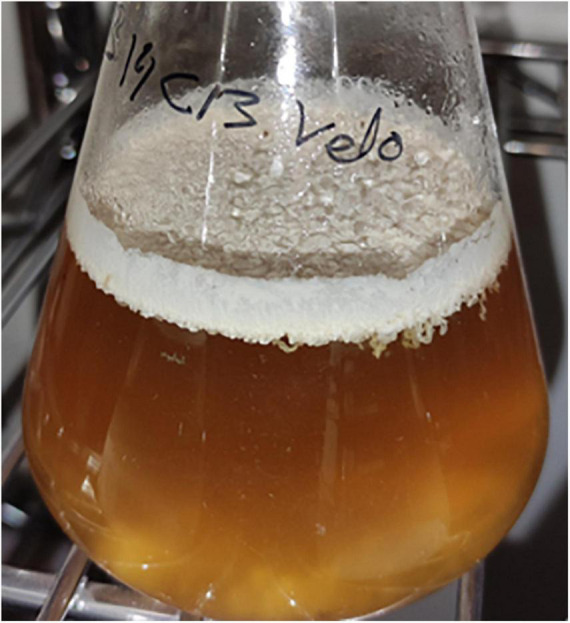
Biofilm formation in regrowth medium after 10 days (5 days at 28°C and 175 rpm and later under static conditions at 22°C for 5 days).

The flor velum samples for yeast identification were treated directly and after the “regrowth medium” (cultivated in 1:1 YPD:wine) in laboratory conditions. After 5 days of static incubation at 22°C, a flor velum or biofilm formation was observed in regrown samples on the surface of the medium. The biofilms were generally thick at the beginning and less consistent in the following days due to a progressive precipitation (Figure 2).

### Yeast Identification

#### Taxonomy Diversity by Internal Transcribed Spacer-Metabarcoding

Internal transcribed spacer region analysis was carried out in the velum samples of solera 1, solera 2, and criadera 1 barrels. In those flor samples in which DNA was extracted directly, *S. cerevisiae* was the only species identified; however, two non-*Saccharomyces* species of the genus *Pichia* were reported predominant over *S. cerevisiae* when samples were previously regrown (Table 1).

**TABLE 1:** Species identified by ITS-metabarcoding, localization, and frequency (%) in each sample.

	*Saccharomyces cerevisiae*	*Pichia manshurica*	*Pichia membranifaciens*
Solera 1 regrowth	22.90	74.72	2.38
Solera 1	100.00	0.00	0.00
Solera 2 regrowth	13.20	83.88	2.90
Solera 2	100.00	0.00	0.00
Criadera 1 regrowth	100.00	0.00	0.00
Criadera 1	100.00	0.00	0.00

#### Matrix-Assisted Laser Desorption/Ionization Time of Flight Mass Spectrometry Identification

All yeast isolates from solera or criadera without regrowth treatment were identified as *S. cerevisiae* as expected; however, in some of the regrowth samples, non-*Saccharomyces* species were found (Table 2). Differences in the identified yeasts were reported depending on the barrel sampled.

**TABLE 2:** Species identified by MALDI-TOF MS, localization, and frequency (%) in each sample.

	*Saccharomyces cerevisiae*	*Pichia manshurica*	*Wickerhamomyces anomalus*	*Trichosporon ashaii*	*Sp. 1*	*Sp.2*	*Candida guillermondii*
Solera 1	20.00	0.00	0.00	0.00	80.00	0.00	0.00
Solera 2	20.00	72.00	7.00	0.00	0.00	1.00	0.00
Solera 3	100.00	0.00	0.00	0.00	0.00	0.00	0.00
Solera 4	100.00	0.00	0.00	0.00	0.00	0.00	0.00
Solera 5	100.00	0.00	0.00	0.00	0.00	0.00	0.00
Solera 6	100.00	0.00	0.00	0.00	0.00	0.00	0.00
Solera 7	0.00	0.00	0.00	100.00	0.00	0.00	0.00
Criadera 1	100.00	0.00	0.00	0.00	0.00	0.00	0.00
Criadera 2	100.00	0.00	0.00	0.00	0.00	0.00	0.00
Criadera 3	100.00	0.00	0.00	0.00	0.00	0.00	0.00
Criadera 4	13.30	0.00	0.00	0.00	0.00	0.00	86.70
Criadera 5	100.00	0.00	0.00	0.00	0.00	0.00	0.00
Criadera 6	40.00	60.00	0.00	0.00	0.00	0.00	0.00

#### Flor Biofilm Typing by Non-*Saccharomyces* Yeasts and *Saccharomyces cerevisiae*

**FIGURE 3:**
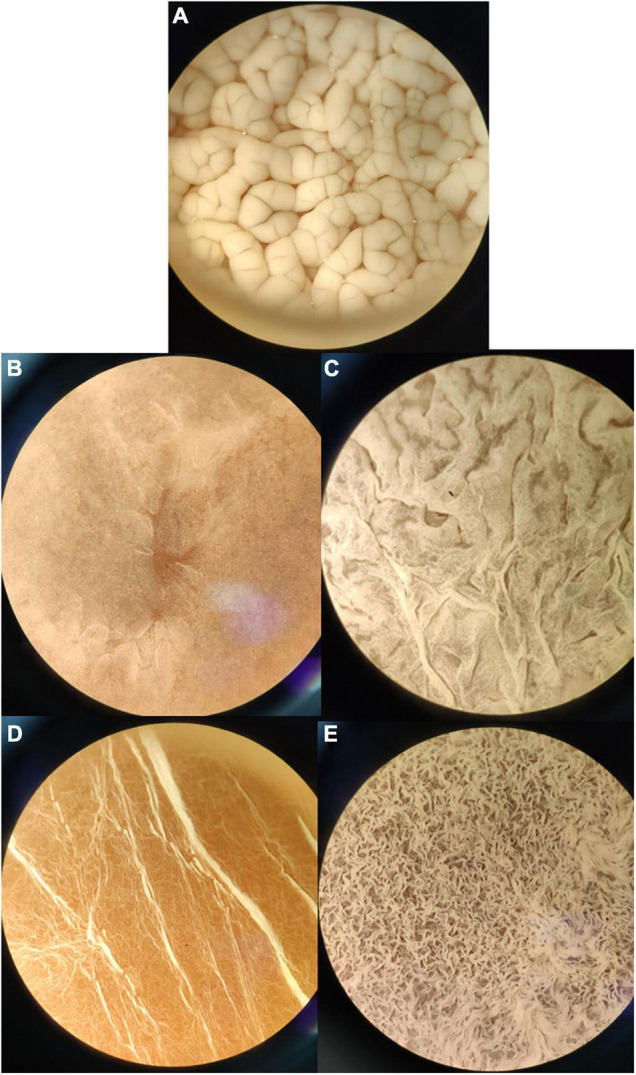
Biofilm formation by different axenic yeast cultures at 10× once the biofilm covered the whole medium surface. **(A)** *S. cerevisiae* flor in wine after 25 days of inoculum; **(B)** *Pichia manshurica* in YPD after 5 days; **(C)** *Trichosporon ashaii* in YPD after 5 days; **(D)** *W. anomalus* in YPD after 5 days. **(E)** Non-*Saccharomyces sp.* in YPD after 5 days.

**FIGURE 4:**
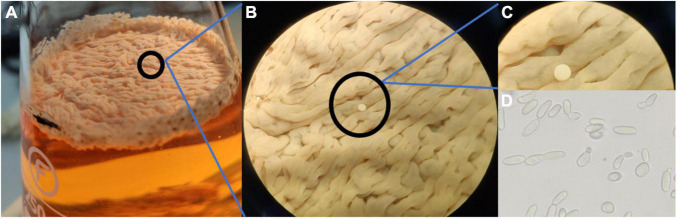
Colony of a putative non-*Saccharomyces* yeast growing over flor yeast velum. **(A)** Sample of velum cultured in wine after 30 days at laboratory; **(B,C)** View of a non-*Saccharomyces* yeast growing on the flor with stereoscopic microscope at 10× and 30×; **(D)** Morphology of the non-*Saccharomyces* yeast at 400×.

It was observed that the isolated and identified non-*Saccharomyces* yeasts were not able to form flor biofilm in wine, whereas they form biofilm in YPD broth or regrowth media. This may be due to the presence of a fermentable carbon source when adding YPD. The isolated yeasts *S. cerevisiae*, *Pichia manshurica*, *Wickerhamomyces anomalus*, *Trichosporon ashaii*, and a non-identified yeast called “sp1” form different types of biofilm (Figure 3). Flor samples obtained from wineries cultivated in the laboratory form biofilm all over the wine surface after 25 days of biological aging, and were observed under stereoscopic microscope (10× and 30×). Interestingly, colonies were observed growing over some biofilms when the samples extracted from the barrels were cultivated in wine (Figure 4). Some of these colonies were isolated onto YPD plates and cells visualized under 400× microscopy, showing an elongated structure and pleomorphism, similar to the non-*Saccharomyces* yeasts previously isolated.

### Oenological Analysis

Multiple comparison analysis analysis identifies significant differences with Bonferroni’s test at a confidence level of 95%. Table 3 shows the average, standard deviations and HG established in relation to the presence/absence of non-*Saccharomyces* yeasts in the solera and criadera samples. A total of 19 variables were analyzed, and only 1-propanol displayed four HGs, which agrees with the four groups established by MCA (Table 3). Also, there are six variables (acetaldehyde, 1,1-diethoxyethane, isobutanol, acetoin, ethyl lactate, and 2,3-butanediol) that display three HGs indicating that at least two wines show no difference between them. Table 3 shows that criadera wines have the same HG in all variables, including 1-propanol concentration. Solera groups show no differences between 12 of the variables.

**TABLE 3:** Means and standard deviations for oenological variables.

	Solera w/o non-S*ch*	Solera w non-*Sch*	Criadera w/o non-*Sch*	Criadera w non-*Sch*
**Fraction or compound**				
Acetaldehyde	235.25 ± 30.13^c^	145.5 ± 7.67^b^	58.48 ± 0.89^a^	72.94 ± 14.60^a^
Ethyl acetate	21.89 ± 0.31^a^	23.13 ± 0.56^a^	73.79 ± 1.04^b^	74.08 ± 3.014^b^
1,1-Diethoxyethane	19.20 ± 2.19^c^	5.32 ± 4.61^b^	0.00^a^	0.00^a^
Methanol	100.18 ± 2.6^a^	93.42 ± 41.8^a^	79.55 ± 5.4^a^	83.41 ± 13.29^a^
1-Propanol	63.15 ± 2.14^d^	60.21 ± 3.82^cd^	55.44 ± 1.26^a^	56.86 ± 0.69^ab^
Isobutanol	79.73 ± 0.57^c^	78.89 ± 1.71^c^	45.87 ± 0.13^a^	48.38 ± 0.19^b^
Isoamyl alcohol	394.76 ± 4.78^b^	401.04 ± 7.68^b^	322.79 ± 2.38^a^	328.72 ± 0.66^a^
Acetoin	68.9 ± 11.8^c^	55.88 ± 4.71^b^	14.77 ± 1.56^a^	14.75 ± 2.01^a^
Ethyl lactate	112.87 ± 19.27^b^	48.60 ± 3.62^a^	206.69 ± 9.93^c^	215.03 ± 15.69^c^
2,3-butanediol (l + m)	1571.02 ± 359^bc^	1694.21 ± 56.38^c^	1215.94 ± 112.49^ab^	1191.86 ± 103.63^b^
Diethyl succinate	102.85 ± 37.79^b^	95.17 ± 8.22^b^	52.36 ± 4.83^a^	52.97 ± 3.64^a^
2-phenylethanol	66.89 ± 11.01^b^	71.97 ± 4.00^b^	47.56 ± 11.03^a^	41.16 ± 3.44^a^
Glycerol	1425.08 ± 178.80^a^	1146.67 ± 11.18^a^	8646.03 ± 641.07^b^	8029.87 ± 304.77^b^
Ethanol (% v/v)	15.17 ± 0.2^a^	14.54 ± 0.2^a^	14.99 ± 0.2^a^	14.89 ± 0.2^a^
Relative density	0.9867 ± 0.0007^a^	0.9867 ± 0.00^b^	0.9877 ± 0.00^b^	0.9877 ± 0.00^b^
Volatile acidity (g/L)	0.13 ± 0.01^a^	0.18 ± 0.00^b^	0.34 ± 0.01^c^	0.36 ± 0.01^c^
Titratable acidity (g/L)	4.01 ± 0.27^a^	4.01 ± 0.00^a^	4.49 ± 0.06^b^	4.74 ± 0.21^b^
pH	3.16 ± 0.03^a^	3.2 ± 0.00^b^	3.21 ± 0.01^b^	3.21 ± 0.00^b^
Free SO_2_ (mg/L)	7.8 ± 0.77^a^	10 ± 0.00^ab^	12.3 ± 1.52^bc^	14.00 ± 2.00^c^

*Volatile aroma compounds and polyols of wines sampled.*

*Concentration in volatile compounds and polyols are expressed as mg/L.*

*±, Standard deviation; abcd, Homogeneous groups among the seven groups of sampling.*

*Different letters denote significant differences at the 95% level in Bonferroni’s test.*

*The alphabetic order of letters indicates the sequence of content.*

*Solera w/o non-Sch, solera without non-Saccharomyces; Solera w non-Sch, solera with non-Saccharomyces; Criadera w/o non-Sch, criadera without non-Saccharomyces; Criadera w non-Sch, criadera with non-Saccharomyces.*

**FIGURE 5:**
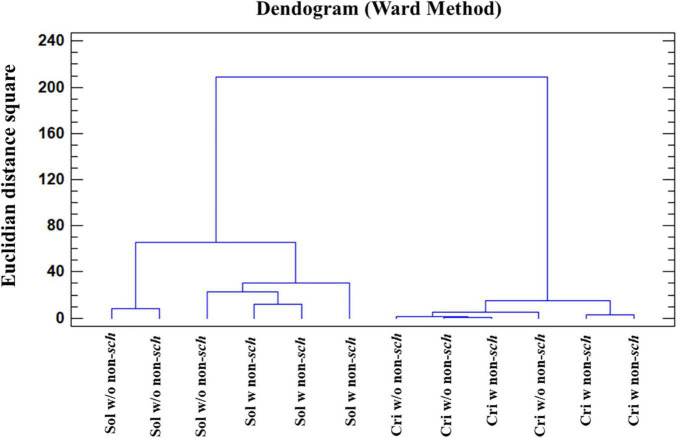
Cluster analysis of wines with different aging times and the presence of non-*Saccharomyces* yeast. The analysis was carried out with all studied variables. Sol w/o non-*sch*, Solera wine without non-*Saccharomyces*; Sol w non-*sch*, Solera wine with non-*saccharomyces*; Cri w/o non-*sch*, Criadera wine without non-*Saccharomyces*; Cri w non-*sch*, Criadera wine with non-*Saccharomyces*.

AC analysis identifies those samples showing a significant difference by means of the squared Euclidean distance, as a measure of the proximity between two samples and the method of Ward as a clustering rule. Considering that the chemical composition of the samples is affected by the aging time (criadera and solera) and by the presence/absence of non-*Saccharomyces* yeasts, AC using the 19 variables was carried out. Firstly, the AC grouped the samples in the function of biological aging time, forming one group for the solera and a second one for the criadera sample. Samples with and without non-*Saccharomyces* yeasts are mixed in both cases, being closer in the criadera samples (Figure 5). If the seven variables with the most HG (Table 3) were selected, the analysis groups samples in the function of the biological aging, but this time, solera samples are differentiated in the function of the presence/absence of non-*Saccharomyces* yeasts.

**FIGURE 6:**
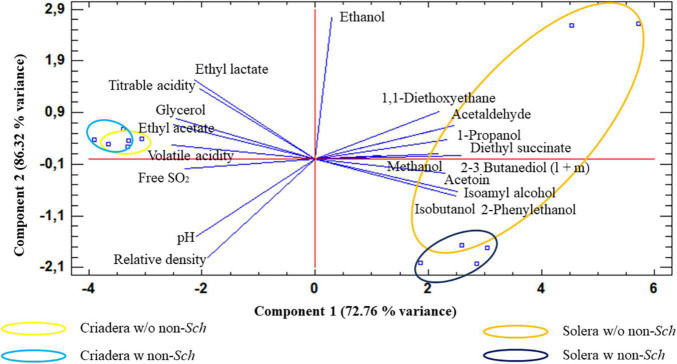
Principal component analysis of wines with different aging times and the presence of non-*Saccharomyces* yeast. The analysis was carried out with all studied variables. Solera w/o non-*sch*, Solera wine without non-*Saccharomyces*; Solera w non-*sch*, Solera wine with non-*Saccharomyces*; Criadera w/o non-*sch*, Criadera wine without non-*Saccharomyces*; Criadera w non-*sch*, Criadera wine with non-*Saccharomyces*.

Principal component analysis analysis agrees with the AC (Figure 6), when the samples were analyzed based on all variables. Two principal components explain the 86.32% of total sample variance. The biplot shows that criadera samples are very similar to the other, while solera samples are grouped based on the presence of non*-Saccharomyces* yeast. The biplot shows that this difference is mostly caused by the ethanol concentration.

Ethanol, ethyl lactate, pH, and titratable acidity were the parameters that contribute most to the variance.

## Discussion

Internal transcribed spacer-metabarcoding techniques are useful to study the microbiota diversity in winemaking processes such as alcoholic or malolactic fermentation without the risk of ignoring microorganisms that are not cultivable under laboratory conditions (Bokulich et al., 2016; Mezzasalma et al., 2017; Stefanini and Cavalieri, 2018). We could confirm through the application of diversity and metataxonomy identification analyses in the biological aging process that *S. cerevisiae* is the most dominant yeast species in the flor microbiota. However, the presence of non-*Saccharomyces* under flor velum formation conditions after a regrowth treatment of samples indicates that the techniques employed have certain limitations when the microorganisms are present in low proportions. The large number of extracellular proteins and other compounds present in the biofilm (Moreno-García et al., 2017) can hinder the DNA extraction and affect the whole study.

On the other hand, MALDI-TOF MS has been very useful to identify most of yeast isolates in a quick and economical manner; however, as it is observed in Table 2, it was not possible to identify all isolates, maybe due to a lack of entries in the MBT Compass Library. It will be interesting to see if the two missing species can be identified using traditional methods (D1:D2 and/or ITS) sequencing in future investigations.

Regrowth process through wine + YPD broth (1:1) seems to be a good step to conduct before identification. This will reveal the presence of non-*Saccharomyces* yeasts in flor biofilms that will otherwise remain undetected. This technique has not been used before in other studies and may be a good option to find those yeasts present in low proportion in the criaderas–solera system. When the same sample is analyzed by both technologies without regrowth, the same results are obtained (100% of *S. cerevisiae* presence), but regrowth samples differ in the species identification. MALDI-TOF MS provided a higher number of species than ITS-metabarcoding.

The presence of non-*Saccharomyces* yeasts in Sherry wine is scarce, in many cases, less than 1% (Ruiz-Muñoz et al., 2020). In fact, when flor is directly plated on YPD agar, no colonies of these yeast species are obtained, showing up only (and in high concentrations) in the presence of a fermentable carbon source in liquid media. Hence, it is justified that they do not appear in large numbers in criaderas–solera biofilms, where the sugar level is close to 0.

The metabolomic study revealed no significant differences between wines with non-*Saccharomyces* and wines without from the criadera stage with most of the variables grouped in the same HG (Table 3). Significant differences in the solera samples are mainly due to ethyl lactate, pH, ethanol, and titratable acidity, the last three variables being easily modifiable and very dependent of the enological practices (Moreno-García et al., 2013). For this reason, we think that non-*Saccharomyces* yeasts present in the biofilms have low influence in the Sherry wine metabolome, unlike the high impact during the alcoholic fermentation of grape must (Jolly et al., 2014). *Pichia* genus, *W. anomalus*, and *Candida guillermondii* are usually detected in high proportions in grape must fermentation or other fermented beverages, such as beer or cider, being progressively replaced by *S. cerevisiae* when ethanol concentrations rise above 4% (Vicente et al., 2021).

It is the first time that *T. ashaii* has been identified in Sherry wines. There is an evidence of its presence in fermented beverages from African and Asian rice wines (Attchelouwa et al., 2018; Wang et al., 2020). In winemaking, *T. ashaii* extracellular beta-glucosidases was studied to enhance aroma in Cabernet Sauvignon by Wang et al. (2012).

*Wickerhamomyces anomalus* has been previously isolated from the flor yeast velum of criadera and solera, *P. manshurica* from criadera, and *P. membranifaciens* was found in sobretablas; all these non-*Saccharomyces* were found in Jerez (Ruiz-Muñoz et al., 2020), but this is the first time that all these yeasts were isolated and identified from solera (*P. manshurica, P. membranifaciens*, and *W. anomalus)* and criadera (*P. manshurica*) in the Montilla-Moriles region.

Further, this is the first report of *C. guillermondii* in the criadera. This species is usually found in red wines and recently applied with *S. cerevisiae* to improve wine color, due to hydroxycinnamate decarboxylase enzymatic activity (Benito et al., 2011). This yeast is also reported to be able to reduce the final ethanol concentration by around 2% when compared to *S. cerevisiae* control (Varela and Varela, 2019).

The main source of non-*Saccharomyces yeasts* found in criadera could be the sobretablas wine, a young wine used to refill higher scales of criadera stage, because the barrels in which they were located were refilled (“rocio”) 1 month before samples were taken, and other authors have found non-*Saccharomyces* species being dominant in this wine (Ruiz-Muñoz et al., 2020).

Solera barrels had not been refilled for more than a year, and it is a six criaderas wine, so the wine used to refill these barrels it is an old wine too; for this reason, we agree with Ruiz-Muñoz et al. (2020) who described these non-*Saccharomyces* as adapted yeasts to these stressful environments. Some isolated species are able of biofilm forming in fermentable media (Figure 3) but the presence of *C. guillermondii* and the non-identified species 2 in samples, both unable of biofilm forming even in YPD or regrowth media, and the presence of non-*Saccharomyces* colonies over the flor yeast velum of *S. cerevisiae* as we saw in laboratory (Figure 4), lead us to hypothesize that they grow consuming the extracellular proteins and other nutrients from dead flor yeasts. Further studies are required to confirm this hypothesis.

The high concentration of acetaldehyde and ethanol (and maybe other compounds) could have caused mutations in the genome of these non-*Saccharomyces* yeasts in a similar way as in *S. cerevisiae* flor strains (Ristow et al., 1995).

Further studies are needed to understand if these yeasts have an impact on the quality of Sherry wines, but it seems that they have higher ethanol tolerance than other strains isolated from different environments. Therefore, these yeasts may be of interest in wine production because they would produce important metabolites for longer times due to their ethanol tolerance. Killer toxin production by the non-*Saccharomyces* yeasts identified, such as the one produced by yeasts of the *Pichia* genus (Vicente et al., 2021) could be a potential way to reduce SO_2_ levels in wine as market demands (Zara and Nardi, 2021).

In conclusion, ITS-metabarcoding and MALDI-TOF MS investigations reveal the existence of eight different yeast species in Montilla-Moriles Sherry wine, two of which are completely new in the criaderas and solera system. These two technologies were able to identify non-*Saccharomyces* yeasts if a regrowth step of the flor velum yeast is performed before the analysis, but they obtain different results in the same sample. We believe that MALDI-TOF MS identification is an easy, cheap, and quick way to identify yeasts from velum samples, as long as a wider database to compare the peptide mass fingerprint is available. On the other hand, the regrowth treatment may be a method to search for non*-Saccharomyces* yeasts in hard environments such as the biological aging of these special wines that could have a biotechnological potential in fields such as food microbiology and biofuels production.

## Data Availability Statement

The data presented in the study are deposited in the Sequence Read Archive (SRA) repository (NCBI), accession numbers SRR17177917–SRR17177922 (https://dataview.ncbi.nlm.nih.gov/object/SRR17177922; https://dataview.ncbi.nlm.nih.gov/object/SRR17177921; https://dataview.ncbi.nlm.nih.gov/object/SRR17177920; https://dataview.ncbi.nlm.nih.gov/object/SRR17177919; https://dataview.ncbi.nlm.nih.gov/object/SRR17177918; and https://dataview.ncbi.nlm.nih.gov/object/SRR17177917).

## Author Contributions

JC-P conducted the study, analyzed the experimental data, and drafted the manuscript along with JM-G. JuM helped to analyze and review the experimental data, participated in the selection and interpretation of the statistical analysis applied, and coordinationed the study. JCM and TG-M coordinated the work, critically reviewed the manuscript prior to submission, and secured the acquisition of funds. All authors approved the final version of the manuscript.

## Conflict of Interest

The authors declare that the research was conducted in the absence of any commercial or financial relationships that could be construed as a potential conflict of interest.

## Publisher’s Note

All claims expressed in this article are solely those of the authors and do not necessarily represent those of their affiliated organizations, or those of the publisher, the editors and the reviewers. Any product that may be evaluated in this article, or claim that may be made by its manufacturer, is not guaranteed or endorsed by the publisher.
